# Development of dual enzyme responsive molecular AND logic gate

**DOI:** 10.55730/1300-0527.3329

**Published:** 2021-12-24

**Authors:** Sündüs ERBAŞ ÇAKMAK

**Affiliations:** 1 Department of Molecular Biology and Genetics, Konya Food and Agriculture University, Konya, Turkey; 2 Biotechnology Graduate Program, Konya Food and Agriculture University, Konya, Turkey

**Keywords:** Molecular logic gates, enzyme sensor, fluorescence, resorufin

## Abstract

Molecular logic gates are information processing devices that can respond to environmental signals and produce a readable output in response through Boolean logic operations. Molecules with these properties have been used to build smart sensors and therapeutic agents. In this work, dual enzyme-responsive molecular AND logic gate is developed with the intention to discriminate various combinations of enzyme level and/or activity. A resorufin-based sensor is substituted with self-immolative tyrosinase recognition site, 3-hydroxy benzyl group. The Hydroxyl group is protected with acetyl moiety which decreases the affinity of the enzyme. When both tyrosinase and esterase are present in the solution, the acetyl group is removed by the latter enzyme, allowing the former to recognise the ligand. Oxidation of the ligand by tyrosinase triggers self-immolative cleavage of the substitution, leading to almost 70 fold enhancement in fluorescence. When single enzyme is applied, there is no significant change in the emission intensity overall, an AND logic gate is constructed. Selectivity and Michaelis-Menten kinetics of the sensor is analysed. Smart molecular probes can contribute to the research on the development of biosensors that can discriminate diseases having characteristic combinations of enzyme activities.

## 1. Introduction

Molecules can be functionalized rationally to be responsive to their environments. Various acivatable therapeutics, molecular sensors that produce a readable output signal in the presence of analyte have been widely studied in the literature [1,2]. In the last decade, significant progress in the design of such systems has been taken. Molecules are further modified to have additional features, multianalyte responsiveness. These findings lead to the discovery of a new concept, molecular logic gates. Logic operations are well-defined mathematical operations where inputs and outputs are characterised by certain rules [3]. Analyte level or response output level beyond a certain threshold are accepted as ‘1’ and the absence of them are given the value of ‘0’. Similar to computer algorithms, this digitalized data is used for logic operations. A potential chemical and biological application of these operations with molecular devices is started to be discussed after the introduction of the molecular logic gate by de Silva et. al. [4]. In this pioneering work, the molecule can detect the inputs (acid and sodium ion), and produces an output in the form of fluorescence. Later, the idea is used to develop many different advanced logic gates such as DEMUX, FLIP-FLOP logics as well as integrated logic gates [5]. Some of these logic gates are shown to have potential applications as smart therapeutics or biosensors [6–20]. Recently, our research group designed a molecular keypad lock to show that information processing devices can have selective therapeutic activity in drug-resistant cancer cells, through a sequential analyte responsiveness and molecular encryption [6]. Theranostic approaches, by which a combinatorial imaging and therapeutic effect can be achieved, is also shown to be possible with advanced molecular logic gate operations [7,8].

Enzymes are biological catalysts whose levels and activities are strictly regulated. Depending on the needs of the cells, extracellular and/or intracellular signals, enzymes are produced, degraded, activated or deactivated. Diseases, including cancer, are usually characterised by abnormal level or activity of a group of enzymes [21]. For that, elevated level of enzymes is considered as a disease marker and various molecular sensors are developed to detect enzyme levels [22,23]. Although diseases can be monitored by following the enzyme level, tissue expression of enzymes may also differ in a healthy organism [24]. The enzyme of interest may also be overexpressed in a different tissue under normal conditions. This may lead to misinterpretation of the data. This constitutes a major problem with single enzyme sensing for diagnostic applications, and a direct correlation between enzyme level and the disease status cannot be achieved. For that, more than one enzyme (or analyte) can be monitored to selectively discriminate healthy tissue from the diseased one. Molecular AND logic operations are suitable devices to have this discrimination. When truth tables of this operation are analysed, these devices only produce an output in the presence of all inputs. In other words, a diagnostic signal is produced when all of the disease parameters are sufficiently high. Previously various dual enzyme sensing probes are reported [25–30]. To the best of our knowledge, there is no fluorescent small-molecule probe dual responsive to tyrosinase and esterase enzymes, which is aimed in this work.

**Scheme 1:**
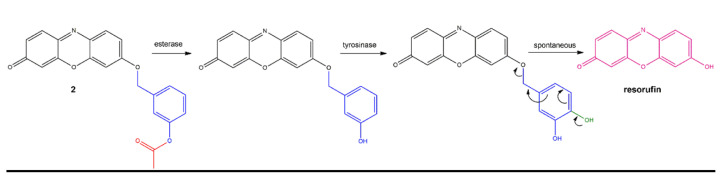
Mechanism of fluorescent resorufin generation from compound 2 in the presence of esterase and tyrosinase enzymes. The presence of ester (red) prevents recognition of the ligand by tyrosinase. Removal of this group exposes tyrosinase binding site, leading to oxidation (green). Oxidized ligand spontaneously dissociate by 1,6-elimination producing fluorescent resorufin.

In the research, an esterase and tyrosinase responsive fluorescent probe 2 is designed as shown in Scheme 1. Resorufin dye is functionalised with tyrosinase enzyme ligand, 3-hydroxy benzyl moiety. This group is known to be oxidized by the enzyme to produce, 3,4-dihydroxy benzyl derivative [31–34]. This self-immolative linker detaches from the parent fluorophore spontaneously by rapid 1,6-elimination reaction, producing fluorescent resorufin (Scheme 1). 3-Hydroxy benzyl recognition unit is preferred because of its stability towards other reactive oxygen species [31]. In this work, phenolic OH is protected with the acyl group to avoid tyrosinase recognition. Upon removal of the acyl group by an esterase, tyrosinase can recognise and oxidise the probe [6]. Therefore, with the action of two enzymes, a fluorescence signal is expected to be produced. Dual enzyme responsiveness is intended to enable accurate discrimination of various phenotypes of diseases for future applications. Tyrosinase enzyme is an oxidative enzyme either catalyses second oxidation of phenolic compounds or produces quinones from diphenols [35]. It is involved in L-DOPA and melanin biosynthesis. Several diseases including Parkinson’s disease and melanoma cancers are reported to show an abnormal level of this enzyme [36, 37]. Esterase enzymes are hydrolytic enzymes responsible for the cleavage of ester bonds. They are known to have roles in drug detoxification and are associated with drug resistance [38]. Therefore, designing a probe responsive to both of these enzymes would enable discrimination of melanoma cancers or Parkinson’s disease that are resistant to traditional chemotherapies. This strategy can be further extended to display response in the presence of various combinations of other enzyme disease markers.

Resorufin is a small fluorescent molecule that has been previously used for many biosensing applications [39, 40]. Functionalization of the OH moiety is known to reduce fluorescence. Once the substituent is removed by the analyte of interest, fluorescence can be reestablished. This TURN-ON fluorescent sensing approach was used to prepare probes for several analytes including enzymes [34, 39–43]. Recently, tyrosinase responsive probe based on resorufin skeleton is also reported with efficient and selective response to this enzyme [34]. In this work, dual responsiveness is generated by hiding the tyrosinase recognition site with acyl group. Acyl groups are previously reported to be removed by the esterase enzymes [6].

## 2. Experimental

General information about the materials, equipment, additional experimental and characterization data are given as supplementary information.

### 2.1. Synthesis

#### 2.1.1. 3-(bromomethyl)phenyl acetate (1)

**Scheme 2:**
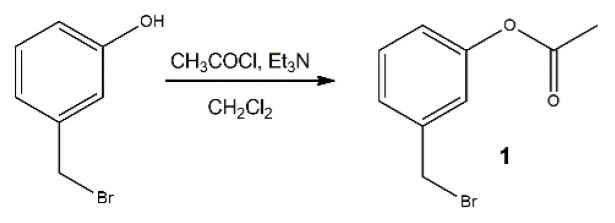
Synthesis of compound 1.

3-Hydroxybenzyl bromide (94 mg, 0.50 mmol) was dissolved in 10 mL dichloromethane. Triethylamine (0.14 mL, 1 mmol) and acetyl chloride (72 μL, 1 mmol) were added (Scheme 2). The reaction was stirred for 16 h at room temperature and then extracted with dichloromethane and water. The organic layer is collected and dried using sodium sulfate. The solution is filtrated to remove sodium sulfate and the solvent was removed using a rotary evaporator. Light yellow oil is obtained as a product. Purification with silica column chromatography results in partial decomposition. For this reason, the crude product is used in the final reaction without further purification (100 mg, 88% yield).

^1^H NMR (400 MHz, CDCl_3_, ppm) δ 7.37 (t, J = 7.8 Hz, 1H), 7.26 (m, 1H), 7.14 (m, 1H), 7.08-6.99 (m, 1H), 4.57 (s, 2H), 2.30 (s, 3H).

^13^C NMR (400 MHz, CDCl_3_, ppm) δ 169.61, 151.02, 139.24, 129.96, 126.14, 121.99, 121.85, 45.71, 21.36.

HRMS (ESI): Theoritical m/z for (M+Na)^+^ is 250.9684 and experimental m/z for (M+Na)^+^ is 250.18276.

#### 2.1.2. 3-(((3-oxo-3H-phenoxazin-7-yl)oxy)methyl)phenyl acetate (2)

**Scheme 3:**
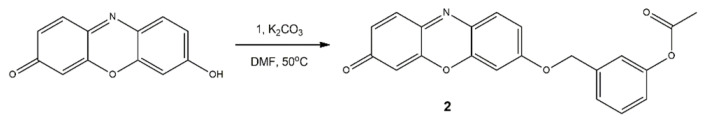
Synthesis of compound 2.

3-(bromomethyl)phenyl acetate (91 mg, 0.40 mmol) and resorufin sodium salt (70 mg, 0.30 mmol) were dissolved in 5 mL dimethylformamide (DMF). Potassium carbonate (138 mg, 1 mmol) was added and the reaction mixture was stirred for 16 h at 50 ^o^C (Scheme 3). The crude product was extracted with dichloromethane and water. The organic layer is collected and dried using sodium sulfate. The solvent was removed using a rotary evaporator. The crude reaction mixture is purified with silica column chromatography using dichloromethane as mobile phase and solid orange product (2) was obtained (60 mg, 55% yield).

^1^H NMR (400 MHz, DMSO-*d*_6_, ppm) δ 7.80 (d, *J* = 8.6 Hz, 1H), 7.54 (d, *J* = 9.4 Hz, 1H), 7.46 (dd, *J* = 8.3, 4.1 Hz, 1H), 7.38 (d, *J* = 7.8 Hz, 1H), 7.26 (s, 1H), 7.21 (s, 1H), 7.17 – 7.06 (m, 2H), 6.79 (d, *J* = 9.5 Hz, 1H), 6.28 (d, *J* = 3.1 Hz, 1H), 5.30 (s, 2H), 2.28 (s, 3H).

^13^C NMR (400 MHz, DMSO-*d*_6_, ppm) δ 185.82, 169.68, 162.61, 151.09, 150.19, 145.83, 145.69, 138.21, 135.41, 134.25, 131.83, 130.19, 128.53, 125.71, 122.17, 121.63, 114.76, 106.14, 101.69, 70.13, 21.34.

### 2.2. Analysis of enzyme-responsiveness

In order to understand enzyme responsiveness of compound 2, 5 mM stock solution was prepared in dimethyl sulfoxide (DMSO). A second solution in PBS buffer (pH 7.4) was prepared with the final concentration of 20 μM. By using the latter solution compound 2 was either incubated alone, in the presence of esterase enzyme, in the presence of tyrosinase enzyme or in the presence of both enzymes for 120 min at 37 °C. Fluorescence spectra were recorded at 30 min intervals using the excitation wavelength of 550 nm. The final concentration of esterase (Esterase Porcine Liver, Enzyme Commission Number 3.1.1.1) and tyrosinase (Mushroom, Enzyme Commission Number 1.14.18.1) enzymes were 2.5 U/mL and 25 U/mL, respectively. In order to allow entry of fresh air, a needle was placed on the lids of reaction vials.

## 3. Results and discussion

**Figure 1:**
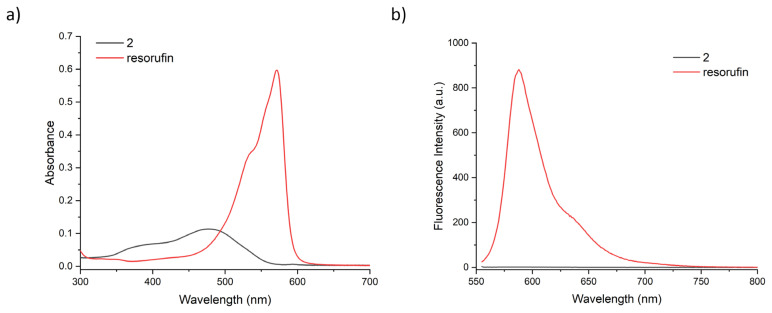
UV-vis absorbance (a) and fluorescence spectra of 20 μM compound 2 (black) and resorufin (red) in PBS buffer, at pH 7.4. Fluorescence spectra are obtained by exciting the sample at 550 nm.

Compound 2 was synthesised in two successive steps and obtained as orange solid with good yields. The compound was characterised using ^1^H NMR, ^13^C NMR, and high-resolution mass spectrometry. Consistent with literature data, O-functionalized resorufin displays a lower absorption at 478 nm and no fluorescence whereas resorufin absorbs at 571 nm and has bright fluorescence at 584 nm in phosphate saline buffer (PBS), at pH 7.4 (Figure 1).The quantum yield and extinction coefficient of the compound were calculated to be 0.04 and 5800 M^−1^cm^−1^ respectively in PBS buffer. Compound 2 has a fluorescence quantum yield much lower than the tabulated value of resorufin (0.97 in water) consistent with the value of similar O-functionalized resorufin derivatives in literature [34, 44].

**Figure 2:**
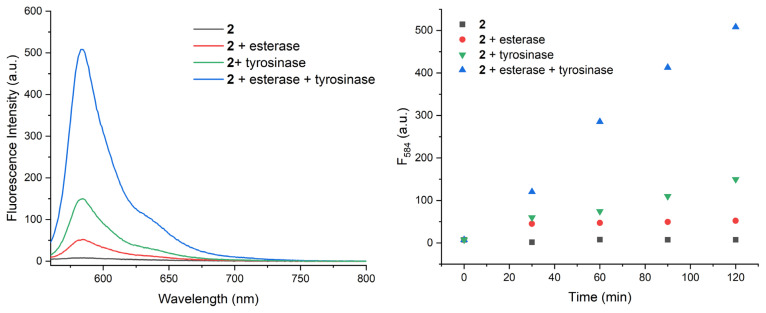
Fluorescence spectra of compound 2, 120 min after incubation with various input combinations (a) and time-dependent change in fluorescence intensity at 584 nm (b). Compound 2 (20 μM, in PBS, pH 7.4) is incubated at 37 °C either alone, with esterase (2.5 U/mL), with tyrosinase (25 U/mL), or with both enzymes for 120 min. Fluorescence at 584 nm is followed to analyse the formation of resorufin. Spectra are recorded by excitation at 550 nm.

To assess the enzyme responsiveness of the probe, compound 2 is incubated alone or with various combinations of inputs and fluorescence spectra are analysed (Figure 2). Almost 70 fold enhancement in fluorescence at 584 nm is detected when both esterase and tyrosinase enzymes are present in the solution. This value is significantly higher than the other input combinations (no enzymes, only esterase or only tyrosinase). Data suggest that in order to obtain fluorescent resorufin, activities of both enzymes are required. A moderate increase in tyrosinase applied samples may indicate slow spontaneous hydrolysis of ester bond in solution. With a separate more concentrated sample (100 μM in PBS buffer), the reaction is repeated with 100 U/mL tyrosinase and 2.5 U/mL esterase enzymes. Change in the solution colour and pink fluorescence of the enzyme-treated sample can be seen with naked eye (Figure S1a and S1b). Low-resolution mass analysis of the dual enzyme treated sample with LCMS proves the formation of resorufin peaks located at 212, corresponding to deprotonated product (Figure S1-c).

**Figure 3:**
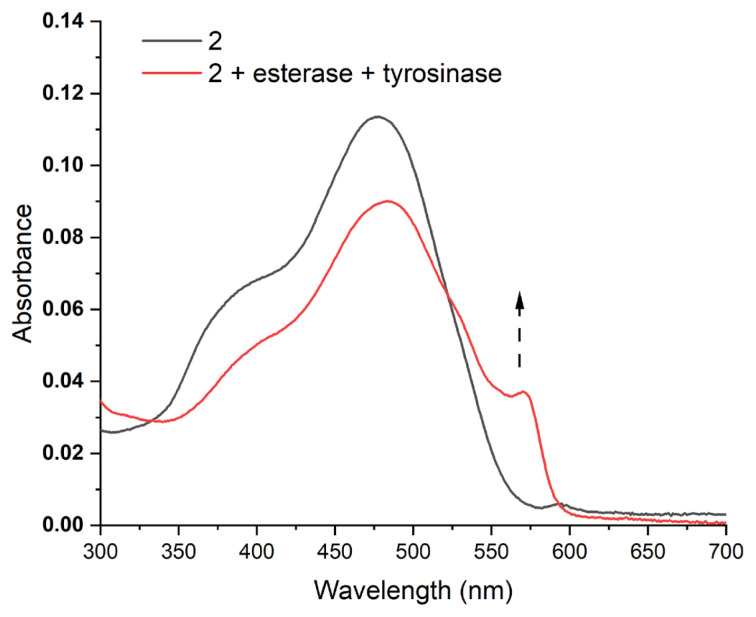
Change in UV-vis absorbance spectra of compound 2 (20 μM, PBS, pH 7.4) in the presence and absence of enzymes. Compound 2 is incubated with 2.5 U/mL esterase and 25 U/mL tyrosinase for 120 min at 37 °C. Peak above 550 nm appears in dual enzyme treated sample, consistent with the formation of resorufin (arrow).

Dual enzyme-treated samples display an increase in the absorption above 550 nm, where free resorufin absorbs (Figure 3). This finding further proves the hypothesis depicted in Scheme 1. Results shown in Figure 2 suggest that when acyl bearing compound 2 is treated with tyrosinase only, fluorescence enhancement is very small. A structurally similar probe that lacks acyl moiety on the structure is reported to be responsive to tyrosinase enzyme [34]. Hence, acyl derivatization seems to prevent enzyme catalysed conversion. To assess this further, Michaelis constant of the enzyme catalysed reaction is calculated. Solutions with different concentrations of compound 2 are treated with 3 U/mL of tyrosinase enzyme and the rate of fluorescence change is determined. Michaelis constant (K_m_) is calculated to be 44.3 μM from the Lineweaver-Burke plot, which is approximately 1.5 fold higher than the tabulated value for a similar probe having ester-free 3-hydroxy phenyl ligand (Figure S2) [34]. This result indicates a lower affinity of the molecule to the enzyme when hydroxyl group is protected by an acyl group. Hence esterase is necessary for the removal of this group to be recognized by tyrosinase. From the same calculations, V_max_ is determined to be 0.37 μM.min^−1^. When probe is incubated in the presence of an abundant serum protein (bovine serum albumin), reducing agents of the body (glutathione, ascorbate) or reactive oxygen species (hydrogen peroxide, hydroperoxide, and superoxide) there is no significant increase in fluorescence indicating the selectivity of the probe towards chosen enzymes (Figure S3).

**Figure 4:**
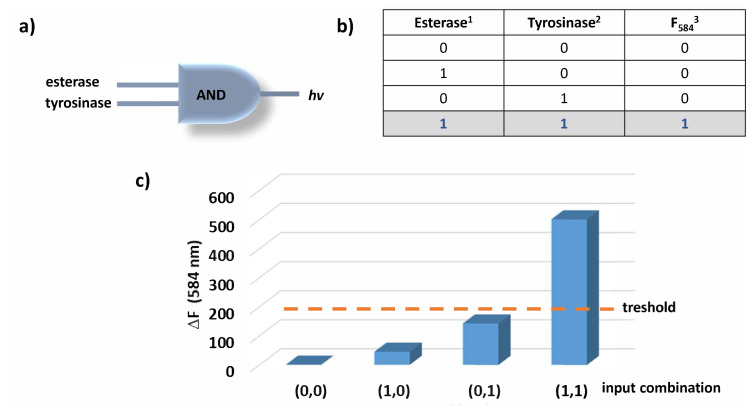
AND logic gate diagram (a) and truth table (b). Threshold for the change in fluorescence is shown with orange dashed line (c). ^1^esterase enzyme is used in 2.5 U/mL concentration. ^2^Tyrosinase enzyme is used in 25 U/mL concentration. ^3^Threshold for fluorescence change is taken to be 150 and the values beyond this value are accepted as 1 in the output column of the truth table.

Considering the data presented so far, compound 2 is shown to display a significant fluorescence enhancement upon incubation with both tyrosinase and esterase enzymes. When the enzymes are introduced alone, the increase is not significant. Altogether, the behaviour of the probe is like an AND logic operation as depicted in Figure 4a,4b. digitalized data is used to construct the truth table of the logic gate (Figure 4b). For the absence of the enzymes, ‘0’ is written and for the presence of the enzymes ‘1’ is written in the first two columns of the truth table. The last column of the table gives the output (fluorescence at 584 nm) and the value is assigned to be ‘1’ if and only if the change in fluorescence is above 150 under experimental conditions. Therefore, the output can only be observed if both enzymes are present in the solution (both inputs are 1).

## 4. Conclusion

Fluorescent molecular sensors are very reliable and sensitive tools for diagnostic applications. Based on the design, sensors can selectively discriminate analytes including enzymes. Expression level and activities of enzymes differ with the progress of certain diseases and this change is usually used for the diagnosis. Since the change in the enzyme level and/or activity is tissue-specific, there should be more than one diagnostic enzyme data, for the accuracy of the analysis. Monitoring more than one disease marker would improve the reliability of the data. In the research presented here, a simple resorufin-based molecular structure is used to overcome this issue. In order to obtain a bright fluorescence, the presence of two enzymes is necessary. This property of the compound makes the molecule an information processing device, with a capacity to detect the signal and produces a readable output as a response. Relatively simple synthetic steps and the efficiency of the enzymatic conversion makes this compound a good candidate for future applications in relevant biomedical areas such as in the diagnosis of drug-resistant melanomas. The same design strategy, hiding the enzyme binding site and exposing it with another analyte, can be used to make other enzyme responsive sensors. This may lead to the production of a library of sensors discriminating various diseases with different enzyme combinations. As conclusion, the dual enzyme responsive molecular logic gate presented here can be further modified to have useful applications in the future.

## Supporting Information

**Development of Dual Enzyme Responsive Molecular AND Logic Gate**

Sundus Erbas-Cakmak^1,2,*^

^1^Department of Molecular Biology and Genetics, Konya Food and Agriculture University, Konya, Turkey

^2^Biotechnology Graduate Program, Konya Food and Agriculture University, Konya, Turkey[Table t1-turkjchem-46-2-567]

**Table t1-turkjchem-46-2-567:** Table of Contents

1. General Information	3
2. Additional Experimental Procedures	4
3. Additional Figures	6
4. References	10

## 1. General Information

All reagents and solvents were purchased from commercial sources and used without further purification. Column chromatography was carried out using silica stationary phase (230–400 mesh, SiliCycle Inc., Canada). Analytical thin layer chromatography was performed on 0.25 mm thick precoated silica gel plates (60F254, Merck, Germany). Esterase enzyme is obtained from Sigma-Aldrich (Porcine Liver Esterase, Enzyme Commission Number 3.1.1.1) and tyrosinase enzyme was purchased from Sigma (mushroom tyrosinase, Enzyme Commision Number 1.14.18.1). Compounds were visualized under UV light. All ^1^H NMR ad ^13^C NMR spectra were recorded on a Varian Inova instrument (400 MHz) at Selçuk University and Ataturk University respectively. Chemical shifts (δ) are reported in parts per million (ppm) and referenced to the residual solvent peak. Coupling constants (*J*) are reported in hertz (Hz). Standard abbreviations indicating multiplicities are given: b = broad, d = doublet, m = multiplet, s = singlet, t = triplet. High-resolution mass spectrometry was carried out using Agilent 6530 Accurate-Mass Q-TOF LC/MS of the Eastern Anatolia Advanced Technology Research and Application Centre (DAYTAM, Erzurum, Turkey). UV-Vis Absorbance and Fluorescence Spectrometry (Agilent, Varian) of Konya Food and Agriculture University are used for spectroscopic analysis. LC-MS data were recorded using Schimadzu LCMS-2020 Single Quadrupole Liquid Chromatography Mass Spectrometer located at Konya Food and Agriculture University.

## 2. Additional Experimental Procedures

### 2.1. Calculation of Quantum yield and Extinction Coefficient

Absorbance and Fluorescence spectra of compounds and reference sample were recorded in PBS buffer and water respectively. As the reference compound Rhodamine 6G is used. Tabulated quantum yield of this compound is used in calculations (0.97 in water)^1^ using the Formula 1 given below.^2^ Samples are excited at 488 nm.


(Formula 1) $$\text{Q}={\text{Q}}_{\text{R}}\;(\text{I}/{\text{I}}_{\text{R}})*({\text{A}}_{\text{R}}/\text{A})*({\text{n}}^{2}/{{\text{n}}_{\text{R}}}^{2})$$

Integrated areas of fluorescence for sample and reference compound (I and I_R_ respectively) are calculated using Origin software. A and A_R_ are absorbance values at 488 nm for sample and reference compound respectively. n is the refractive index and this value is taken to be 1.333, corresponding to the value for water. Quantum yield of Rhodamine 6G (Q_R_) is taken to be 0.95.

Extinction coefficient is determined using Beer-Lambert formula (Formula 2), taking the length of light path (l) as 1 cm.


(Formula 2) A=ɛ C I

In the Formula 2, C refers to concentration of the sample in PBS buffer, A is the absorbance and ɛ is the extinction coefficient.

### 2.2. Determination of Michaelis Constant and Maximum Rates

Samples containing 10, 20, 30, 40 and 50 μM of compound **2** in the presence of 3U/ml tyrosinase enzyme in PBS buffer (pH 7.4) were incubated at 37°C and fluorescence spectra were recorded. Fluorescence was recorded at 0, 15, 45 and 75 minutes. From the time dependent fluorescence change of the samples, initial rates of reactions were calculated for each sample. The data is plotted as Lineweaver-Burke plot as shown in Figure S2. Linear fitting of the data points gives the equation 1 below. Using the slope and intercept values of the equation, Michaelis constant (K_m_) and maximum rate of enzymatic conversion (V_max_) were calculated. [S] in the equation represents the concentration of compound **2**.


(Equation 1) $$1/{\text{V}}_{0}=({\text{K}}_{\text{m}}/{\text{V}}_{\text{max}})*(1/[\text{S}])+1/{\text{V}}_{\text{max}}$$

### 2.3. Selectivity Experiment

Compound 2 (20 μM) was prepared in PBS buffer (pH 7.4). Various analytes were added at the concentrations relevant to physiological ones and incubated for 120 min at 37°C. Fluorescence spectra were recorded and compared with the spectra of dual enzyme treated Compound 2 as shown in Figure S3. Concentrations of the analytes were 1 mM for glutathione (GSH) and sodium ascorbate, 100 μM for Bovine Serum Albumin (BSA), tert-butyl hydroperoxide (TBHP), hydrogen peroxide, potassium superoxide and finally 25U/ml for tyrosinase and 2.5U/ml for esterase.

## 3. Additional Figures

In order to understand the colour and fluorescence change more obviously with naked eye and by Mass Spectrometry analysis, more concentrated compound **2** was prepared (100 μM in PBS buffer). To this solution, esterase enzyme (2.5 U/ml) and tyrosinase enzyme (100 U/ml) were added. A photo under day light and UV light is taken (Figure S1-a and S1-b respectively). Colour change and generation of bright pink fluorescence is clearly seen in dual enzyme treated samples. LC-MS analysis from enzyme treated samples proves the formation of resorufin with a peak value at 212 corresponding to (M-H)^−^.

To determine the Michaelis constant of tyrosinase enzyme, substrate dependency of rates are plotted in the form of Lineweaver-Burke plot (Figure S2). From the slope and intercept values K_m_ and V_max_ are calculated as described in previous section.

Figure S1 Compound **2** (100 μM) in PBS buffer (pH 7.4) under daylight (a) and under UV light (b). Compound **2** is incubated with esterase (2.5 U/ml) and tyrosinase (100 U/ml) enzymes for 30 min in right samples of each photo. Low resolution mass spectra obtained by LCMS analysis of enzyme treated sample (c) proves the formation of resorufin with m/z of 212 corresponding to (M-H)^−^.

Figure S2 Lineweaver-Burke plot for conversion rate of compound **2** (10–50 μM) in the presence of 3 U/ml tyrosinase enzyme in PBS buffer, at 37°C.

Figure S3 Selectivity of the compound **2** towards various different analytes. Samples contains (1) 100 μM BSA; (2) 1mM GSH; (3) 1 mM sodium ascorbate; (4) 100 μM H_2_O_2_; (5) 100 μM KO_2_; (6) 100 μM TBHP; (7) 25U/ml tyrosinase and 2.5U/ml esterase enzymes. Excitation wavelength is 550 nm.

Figure S4 ^1^H NMR spectrum of compound **1** (400 MHz, CDCl_3_)

Figure S5 ^13^C NMR spectrum of compound **1** (400 MHz, CDCl_3_)

Figure S6 HRMS (ESI) spectrum of compound **1**

Figure S7 ^1^H NMR spectrum of compound **2** (400 MHz, DMSO-d6)

Figure S8 ^13^C NMR spectrum of compound **2** (400 MHz, DMSO-d6)

4. References1

BozdemirOA
Erbas-CakmakS
EkizOO
DanaA
AkkayaEU

Towards Unimolecular Luminescent Solar Concentrators: Bodipy-Based Dendritic Energy Transfer Cascade with Panchromatic Absorption and Monochromatized Emission”

BozdemirOA
Erbas-CakmakS
EkizOÖ
DanaA
AkkayaEU

2011
Angewandte Chemie International Edition 2011
50
10907
10912
10.1002/anie.201104846
219538422

XuW
KongJS
YehYTE
ChenP

Single-molecule nanocatalysis reveals heterogeneous reaction pathways and catalytic dynamics
Nature Materials
2008
7
992
996
10.1038/nmat2319
18997774
